# Low-cost, rapidly-developed, 3D printed *in vitro* corpus callosum model for mucopolysaccharidosis type I

**DOI:** 10.12688/f1000research.9861.2

**Published:** 2017-03-16

**Authors:** Anthony Tabet, Matthew Gardner, Sebastian Swanson, Sydney Crump, Austin McMeekin, Diana Gong, Rebecca Tabet, Benjamin Hacker, Igor Nestrasil

**Affiliations:** 1 CoCreateX, Inc., St. Paul, USA; 2 Precision Horizons, Minneapolis, USA; 3 Department of Pediatrics, University of Minnesota, Minneapolis, USA

**Keywords:** 3D printing, neurodegenerative disease, cell culture, in vitro release, mucopolysaccharidosis, corpus callosum

## Abstract

The rising prevalence of high throughput screening and the general inability of (1) two dimensional (2D) cell culture and (2) *in vitro* release studies to predict *in vivo* neurobiological and pharmacokinetic responses in humans has led to greater interest in more realistic three dimensional (3D) benchtop platforms. Advantages of 3D human cell culture over its 2D analogue, or even animal models, include taking the effects of microgeometry and long-range topological features into consideration. In the era of personalized medicine, it has become increasingly valuable to screen candidate molecules and synergistic therapeutics at a patient-specific level, in particular for diseases that manifest in highly variable ways. The lack of established standards and the relatively arbitrary choice of probing conditions has limited *in vitro *drug release to a largely qualitative assessment as opposed to a predictive, quantitative measure of pharmacokinetics and pharmacodynamics in tissue. Here we report the methods used in the rapid, low-cost development of a 3D model of a mucopolysaccharidosis type I patient’s corpus callosum, which may be used for cell culture and drug release. The CAD model is developed from *in vivo* brain MRI tracing of the corpus callosum using open-source software, printed with poly (lactic-acid) on a Makerbot Replicator 5X, UV-sterilized, and coated with poly (lysine) for cellular adhesion. Adaptations of material and 3D printer for expanded applications are also discussed.

## Introduction

Mucopolysaccharidosis (MPS) is a spectrum of inheritable conditions involving the accumulation of glycosaminoglycans (GAGs) following disruption of key lysosomal enzymes, which in turn leads to complications on a cellular, tissue, and organ level ^1^. In MPS type I (MPS I), which is characterized by a deficiency of the enzyme α-L-iduronidase, brain MRI scans reveal thinning of white matter and lesions within the periventricular area and especially the corpus callosum (CC) ^2^. The CC is the largest white matter structure in the brain, with more than 300 million axonal projections, and it interconnects the left and right hemispheres ^3^. MPS I leads to patient-specific, irregular white matter density and geometry in the CC. Current treatment for MPS include enzyme replacement therapy (ERT) and hematopoietic stem cell transplantation (HSCT). ERT has been shown to ameliorate MPS symptoms, yet does not prevent disease progression ^4^, owing in part to poor bioavailability. HSCT has been shown to improve cognitive development. Donors, however, can be hard to find unless umbilical cord blood is available; the procedure also has significant health risks ^5, 6^. As such, more research into potential targets and drug delivery excipients, which can provide tunable release kinetics, is needed to develop a library of promising treatment options. Additionally, owing to the highly patient-specific deterioration of cerebral white matter, patient-specific identification of synergistic drug combinations and optimal drug release kinetics can enable a more personalized medicine approach to treat MPS in the future.

Traditional methods of screening use two-dimensional (2D) cell culture to study biochemical pathways and targets in cells. Yet, 2D designs of traditional cell cultures fail to account for complex cell-cell and/or cell-matrix interactions. There has been a growing literature that demonstrate the importance of three-dimensional (3D) environments in expressing phenotypes, genes, and proteins at levels found *in vivo* and not otherwise seen in 2D models ^7– 9^. 2D i *n vitro* drug release studies of promising therapeutic targets are generally limited to providing qualitative insight into *in vivo* release behavior. Seemingly arbitrary choices in probing conditions such as material volume, material surface area, supernatant volume, and rotator conditions, hinders quantitatively rigorous conclusions of mass transfer, pharmacokinetic, and pharmacodynamics properties to be made from benchtop measurements. These in tandem demonstrate a pressing need for the use of 3D disease models as a more representative *in vitro* system. Here, we describe an inexpensive and fast method of developing such patient specific 3D models.

## Methods

**Figure 1.**
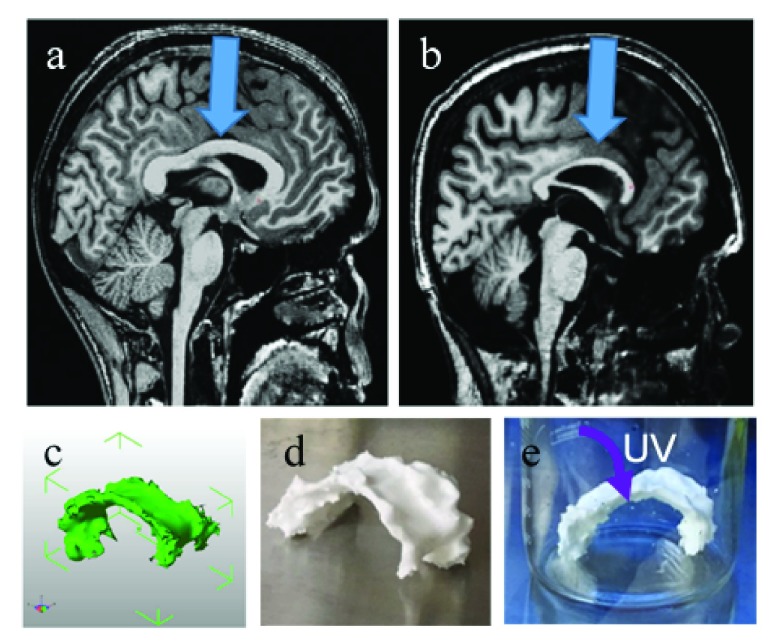
3D models and prints. ( **a**– **b**) T1-weighted brain MRI with resolution of 1×1×1 mm, midsagittal slice, arrow pointing at corpus callous in ( **a**) healthy control and ( **b**) MPS I subject. ( **c**) CAD image of MPS I corpus callosum taken at five adjacent slices in each hemisphere. ( **d**) 3D printed MPS I corpus callosum with poly(lactic-acid) on a Makerbot Replicator 5X. ( **e**) UV sterilizing the print overnight.

The 3D brain MRI scans of a 20-year-old male subject with MPS I and an age-matched healthy male control were manually traced to obtain a 3D structure of the corpus callosum (CC). The 3D model was printed on a Makerbot Replicator 5X, sterilized ( Figure 1), and could be used for cell culture or *in vitro* release studies. The de-identified MRI scans were obtained as Digital Imaging and Communications in Medicine (DICOM) files ( Dataset 1 ^11^). The CC was traced on the mid-sagittal slice and five adjacent slices in each hemisphere using open source InVesalius 3 ( http://www.cti.gov.br/invesalius/, RRID: SCR_014693). Alternatively, OsiriX 8.0.1 software ( http://www.osirix-viewer.com/, RRID: SCR_013618) may also be used. In some cases, data is obtained as a NIfTI-1file with the extension .nii; these files may also be used. MRICron 1.0 ( http://people.cas.sc.edu/rorden/mricron/index.html) RRID:SCR_002403 or mri_convert 1.0 ( https://surfer.nmr.mgh.harvard.edu/fswiki/mri_convert) software packages may be used to convert between DICOM and NIfTI-1. The software was then used to render the scans into a single .STL file ( Dataset 2 ^12^). The 3D model of the CC was loaded into MakerBot Desktop v. 3.6.0.78 ( https://www.makerbot.com/download-desktop/) and printed on a MakerBot Replicator 5X with poly(lactic acid) at a resolution of 0.2 mm, maintaining life-size dimensions. Stratasys post-processing fluid was optionally used to remove any support material. The 3D printed structures were rinsed with a 70% ethanol/water solution and UV-sterilized overnight. The prints were then coated with polylysine (Sigma) for cellular adhesion ^10^, by dipping them upside down in a 0.5 mg/mL poly-L-lysine solution for at least 10 minutes. Only the top of the surface was dipped ( Figure 1d), as this was the area of interest where the drug delivery materials would be loaded, but for other applications discussed in the next section, the entire structure can be dipped into ~50 mL of the poly-L-lysine solution for complete cell adhesion on the top and bottom.

For cell culture, this object would be used in a sterile flask, or alternatively, a larger sterilized container. For use as a drug delivery platform, the object could be both be cultured with cells as previously discussed and kept in cell culture media, or used without cells in PBS. A prenteral drug delivery system, such as shear-thinning hydrogels or hydrophobic polymer melts, can be injected with a syringe on top of the 3D model, until the amount of drug loaded is comparable to translational doses, or until the material completely coats the 3D model. When a cell coated surface is used, the drug will be released into the cell media. Conversely, when a cell-free model is used, the drug release kinetics are monitored in PBS, which is less prone to interference. Care must be taken to ensure that the release kinetic probing molecule’s absorbance or emission spectra are not greatly interfered with by cell culture media.

DICOM files for the de-identified MRI scans of the corpus callosum of a MPS I subject Click here for additional data file. Copyright: © 2017 Tabet A et al. 2017 Data associated with the article are available under the terms of the Creative Commons Zero "No rights reserved" data waiver (CC0 1.0 Public domain dedication).

Resulting CAD file from InVesalius 3 software (.STL), used to render the DICOM files in Dataset 1 Click here for additional data file. Copyright: © 2017 Tabet A et al. 2017 Data associated with the article are available under the terms of the Creative Commons Zero "No rights reserved" data waiver (CC0 1.0 Public domain dedication).

## Discussion

This technique uses, but is not limited to, poly(lactic-acid) (PLA), a readily available filament for desktop 3D printers, such as the Makerbot Replicator 5X. PLA has been widely shown to be biocompatible ^13^. Applications of this platform include studying *in vitro* drug release of injectable drug depots for delivery of therapeutics e.g. proteins for enzymatic deficiency disorders, such as MPS, and hydrophobic small molecules for brain cancer. *In vitro* drug release methodology can largely vary release profiles depending on the geometry of the container used. A 15 mL conical tube provides a different area for mass transfer than a 10 cm culture dish or 5 mL glass vial. This 3D modeling platform can potentially offer a more realistic and more standard geometry for monitoring drug release. Additionally, many therapeutic approaches to treat brain cancer and other diseases rely on injecting or implanting material that maintains a high interfacial concentration to improve drug bioavailability and efficacy, such as gold nanoparticle radiosensitizers for radiotherapy ^14, 15^. The drug depot material can be tested *in vitro* on this platform to determine the proper interfacial concentration given to-scale surface area of the tissue, and monitor the duration to which this concentration can be maintained.

Figure 1 (a–b) demonstrates the thinning of the CC in MPS I (b) compared to that of a healthy brain (a). Given the uniqueness of each MPS patient’s brain pathology, density, and geometry, the ability to test the therapeutic window, effectiveness, and optimal drug loading concentration into an injectable drug depot for each specific patient is highly useful. A team of high school and undergraduate students was able to render the CAD file ( Figure 1c) and 3D print on common desktop 3D printers at a low cost ( Figure 1d), suggesting that this culture may be scaled more readily than expensive 3D *in vitro* platforms. This material’s modulus is approximately 3 GPa, several orders of magnitude larger than native tissue. In order to create a 3D cell culture platform which enables cell migration and proliferation within the tissue, a 3D bioprinting approach must be used ^16, 17^. In conclusion, this method’s robustness, ease, and low cost make it adaptable for use for a wide variety of applications in drug delivery, drug discovery, tissue engineering, and stem cell biology.

## Data availability

The data referenced by this article are under copyright with the following copyright statement: Copyright: © 2017 Tabet A et al.

Data associated with the article are available under the terms of the Creative Commons Zero "No rights reserved" data waiver (CC0 1.0 Public domain dedication). 

**Dataset 1**: DICOM files for the de-identified MRI scans of the corpus callosum of a MPS I subject, doi: 10.5256/f1000research.9861.d144327 ^11^

**Dataset 2**: Resulting CAD file from InVesalius 3 software (.STL), used to render the DICOM files in Dataset 1, doi: 10.5256/f1000research.9861.d144328 ^12^

## Ethics

The study protocol involving the brain MRI acquisition was approved by the University of Minnesota IRB committee. Written, informed consent to publish results from MPS patients and healthy volunteers was obtained.
