# Supplementary material for: Low-cost, rapidly-developed, 3D printed in vitro corpus callosum model for mucopolysaccharidosis type I
Source: F1000Res. 2017 Mar 16;5:2811. Originally published 2016 Dec 2. [Version 2] doi: 10.12688/f1000research.9861.2 (PMC5345771; doi:10.12688/f1000research.9861.2)
Supplement: Peer review history

# Peer review: Referee response for version 1

**DOI:** 10.5256/f1000research.10632.r20282

**Published:** 2017-03-06

**Authors:** Siddharth Chanpuriya, Pooja Jambunathan

**Affiliations:** 1 Axalta Coating Systems, Philadelphia, PA, USA; 2 Merck, Kenilworth, NJ, USA

The authors motivate the problem well by summarizing the drawbacks of 2D cell culture in vitro studies to predict in vivo responses in humans.

 3D human cell cultures offers several advantages; here, the authors highlight how, in this era of personalized medicine, 3D platforms can be tailor-made to offer a more realistic approximation of in vivo studies when examining drug release kinetics. Specifically, a 3D printed model of a mucopolysaccharidosis type I patient's corpus callosum is developed from an MRI trace.

 The report is presented clearly and the authors demonstrate how to rapidly develop the aforementioned 3D model and prepare it for drug delivery studies. However, the paper lacks any studies that demonstrably show the advantage of the 3D model. Future work that experimentally establish the effectiveness of 3D models synthesized using the reported method would be useful.

We have read this submission. We believe that we have an appropriate level of expertise to confirm that it is of an acceptable scientific standard.

# Peer review: Referee response for version 1

**DOI:** 10.5256/f1000research.10632.r18886

**Published:** 2017-01-19

**Authors:** Dustin Sprouse

**Affiliations:** 1 Sherwin Williams, Minneapolis, MN, USA

Indeed our society and health care system is moving toward more personalized medicine and care. Currently this is expensive and there is a great need for high throughput screening, lower costs, and 3D human cell culture to verify new drug discovery.

 The authors sum this up well in the title and introduction and the brief follows this idea throughout and provides an example with the rare genetic disorder of mucopolysaccharidosis.

 The paper flows nicely, but it was the methods section that had a lack of details and data and leaves one with more questions than revelations. The data is available in two linked files; however, one would first need the correct software to open them. Thus, I recommend more images/video to convey what the scientists accomplished and show the readers the potential results and outcomes.

 The discussion sums up some important ideas that need to be considered when developing a methodology such as this one. These methods will need to be independently verified and show promising potential prior to a new mass platform. 

 In conclusion, this paper puts forth the ideas of a low cost approach to 3D printing scaffolds for drug release profiles, hopefully leading to faster turnarounds and lower costs of personalized medicine, drug discovery, tissue engineering, and stem cell biology.

I have read this submission. I believe that I have an appropriate level of expertise to confirm that it is of an acceptable scientific standard.
